# Possibilities for Optimization of Industrial Alkaline Steeping of Wood-Based Cellulose Fibers

**DOI:** 10.3390/molecules25245834

**Published:** 2020-12-10

**Authors:** Catharina Fechter, Harald Brelid, Steffen Fischer

**Affiliations:** 1 Institute for Organic Chemistry and Macromolecular Chemistry, Chemisch-Geowissenschaftliche Fakultät, Friedrich-Schiller-University of Jena, 07743 Jena, Germany; 2 Södra Skogsägarna Ekonomisk Förening, 351 89 Växjö, Sweden; harald.brelid@sodra.com; 3 Institut für Pflanzen- und Holzchemie, Fakultät Biologie, Technische Universität Dresden, 01737 Tharandt, Germany; steffen.fischer@tu-dresden.de

**Keywords:** dissolving pulp, cellulose, xylan, viscose, mercerization, steeping, alkali cellulose, R-value, yield

## Abstract

Steeping of cellulosic materials in aqueous solution of NaOH is a common pre-treatment in several industrial processes for production of cellulose-based products, including viscose fibers. This study investigated whether the span of commonly applied process settings has the potential for process optimization regarding purity, yield, and degree of transformation to alkali cellulose. A hardwood kraft dissolving pulp was extracted with 17–20 wt% aq. NaOH at 40−50 °C. The regenerated residue of the pulp was characterized regarding its chemical composition, molecular structure, and cellulose conformation. Yield was shown to be favored primarily by low temperature and secondly by high alkali concentration. Purity of xylan developed inversely. Both purity of xylan and yield varied over the applied span of settings to an extent which makes case-adapted process optimization meaningful. Decreasing the steeping temperature by 2 °C increased xylan content in the residue with 0.13%-units over the whole span of applied alkali concentrations, while yield increased by 0.15%-units when extracting with 17 wt% aq. NaOH, and by 0.20%-units when extracting with 20 wt%. Moreover, the yield-favoring conditions resulted in a narrower molecular weight distribution. The degree of transformation via alkali cellulose to cellulose II, as determined with Raman spectroscopy, was found to be high at all extraction settings applied.

## 1. Introduction

Steeping of cellulosic materials in aqueous solution of NaOH is a common pre-treatment in several industrial processes for production of cellulose based products [1,2]. Volume-wise, the most important process is the viscose process, which mainly produces regenerated cellulose fibers for textile applications. More than 90% of today’s seven million tons of viscose fiber is produced using dissolving pulp derived from wood (DWP) [3]. In viscose production, alkalization (steeping) is carried out in an alkaline aqueous solution containing cellulose pulp. The steeping turns the pulp into highly swollen alkali cellulose (Na-Cell), which, after pressing of the pulp slurry, is subjected to the aging step where oxidation reactions lead to a decrease in the degree of polymerization (DP) of the cellulose. In the aging step, the DP is adjusted to a level suitable for the requirements of the viscose fiber product aimed for. In the following step, Na-Cell is reacted with carbon disulphide to create an alkali soluble cellulose derivative (cellulose xanthate), which can be converted back to cellulose in the form of regenerated cellulose fibers when the cellulose xanthate solution is spun through a spinneret in an acid coagulation bath to shape the desired product [1,2].

The formation of Na-Cell is important for the viscose process since it facilitates the reaction with carbon disulphide in the xanthation step. Moreover, Na-Cell is easy to depolymerize by means of the oxidation that occurs in the aging step prior to xanthation. Changes in the pulp induced through alkalization have been intensively considered in research, cf. e.g., Rydholm (1965), Kolpak et al. (1978), Fink et al. (1982), Fengel and Wegener (1989), Krässig (1993), Sixta (2006a), Mozdyniewicz et al. (2014) and Reyes et al. (2016) [4,5,6,7,8,9,10,11]. When treating pulp with aqueous alkali and transforming the cellulose part into Na-Cell, there are two further processes taking place: swelling of the entire solid polymer structure and the dissolution of polymers with a low DP, i.e., hemicelluloses and low-molecular fractions of the cellulose. Fechter et al. [12] showed that the intensity of the processes of transformation and dissolution depend not only on the temperature and alkali concentration applied, but also on the pulp used.

Regarding the viscose process, the optimal choice of steeping conditions, namely, alkali concentration and temperature, is a balance among swelling, the extent of transformation to Na-Cell, dissolution of short-chained polymers, which are removed with the lye by means of alkaline extraction through pressing, and the processability of both the press lye and the Na-Cell. According to Woodings [2], recent industrial slurry steeping is commonly done in a slurry with 17–19 wt% aq. NaOH at 45–55 °C. A lower lye temperature is known to hamper processability by resulting in more viscous lye and more swollen Na-Cell. Studies from the 1950s and 1960s had a clear tendency to refer to lower industrial steeping temperatures (20–35 °C) [13,14,15,16,17]. Laboratory studies by Reyes et al. (2016), Fechter et al. (2020) and Wyatt (1966), aiming for the optimization of the alkaline stepping, have confirmed that high alkali concentrations give the best alkalization results [11,12,18]. Moreover, laboratory studies have confirmed a positive effect of the lower temperatures used in former times. The early study by Wyatt recommended universally optimal process settings to be 18.3 wt% aq. NaOH at 15 °C under 27 min [18]. A recent study by Reyes et al. found 21 wt% aq. NaOH at 29 °C with reaction time being uncritical for optimal yield and transformation to Na-Cell [11]. Fechter et al. alkalized different pulps at a few process settings with a wide span (10, 14, and 18 wt% aq. NaOH at 20, 35, and 50 °C) [12]. They found that treating the pulps with the highest alkali concentration led to the best alkalization result for yield, purity and transformation. The choice of steeping temperature has been found to be important to balance the yield and the purity of xylan-containing pulps. The lower extraction temperature favored yield but led to lower purity. While Wyatt and Reyes et al. investigated a single pulp, Fechter et al. identified a potential for process optimization in the alkalization step when using different market dissolving pulps [12]. One of the pulps investigated, a short-fiber kraft pulp with a somewhat elevated xylan content, showed the highest potential for optimization of purity toward yield at high alkali concentration when varying extraction temperature. The present study investigated a similar market pulp applying a narrower range of steeping settings that mirrors today’s viscose production conditions (17–20 wt% aq. NaOH at 40–50 °C). Based on the findings of Fechter et al. and using the same methodology and facilities, the aim of this study is to show whether the narrow span of commonly applied settings has the potential for process optimization regarding the purity, the yield, and the degree of transformation to Na-Cell in the alkalization step.

## 2. Results

The pulp investigated, a hardwood kraft dissolving pulp for use in viscose fiber production, was characterized as summarized in Table 1. The fact that the process history and the chemical composition of the pulp used in this present study was almost identical to the reference in Fechter et al. [12] made it possible to further develop the research conducted in that study. However, the molecular weight of the sample deviated from the reference considered in the previous paper mentioned with regard to higher molecular weight (474 mL/g vs. 391 mL/g) and broader molecular weight distribution (higher polymer dispersion index, PDI; 3.3 vs. 2.8). In consequence, the pulp investigated in this study had a lower fraction of low-molecular-weight material (DP < 100) and a higher fraction of high-molecular-weight material (DP > 2000). Nevertheless, both pulps were still in the normal range of a standard kraft dissolving pulp. The present study investigated two independent variables, alkali concentration (NaOH) and extraction temperature T. All experimental data are given in Table 1.

A multiple linear fitting of the data for the response variables residue after alkaline extraction (R-value) and xylan content in the residue was possible according to Equations (1) and (2). The correlation coefficient R^2^ and the estimate of the predictive ability of the model Q^2^ were both high and close to each other for both responses, R-value (0.95 and 0.87, respectively) and xylan content (0.98 and 0.93, respectively). All other measured responses (data from molecular weight distribution and degree of transformation) had R^2^ < 0,4 and a negative Q^2^, which show that no statistically firm modeling could be done.

(1) $$R-\mathrm{value}=91.423+0.464\;\left[\mathrm{NaOH}\right]+0.056\;T-0.008\;\left(\left[\mathrm{NaOH}\right]\times T\right)$$

(2) $$\mathrm{Xylan}\;\mathrm{content}=43.355+0.002\;\left[\mathrm{NaOH}\right]-0.874\;T+0.013\left(\left[\mathrm{NaOH}\right]\times T\right)$$

### 2.1. The Change in Yield of the Pulp Caused by Alkalization

**Figure 1:**
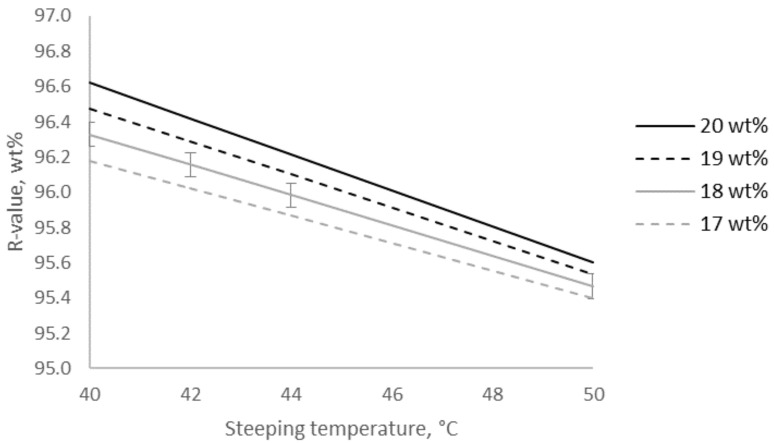
Modeled residue after extraction of hardwood kraft dissolving pulp with aqueous NaOH at different concentrations and temperatures, expressed as R-value, and its RMSE (root mean square error).

It is shown in the literature that yield after alkaline extraction increases with increasing alkali concentration exceeding 10–12 wt% aq. NaOH and increasing temperature, as these conditions lead to a less swollen structure hampering the extraction of polymeric material and leading to higher R-values [7,9,19]. This study quantified the increase of R-value due to increased alkali concentration to 0.2–0.4% units depending on extraction temperature when increasing from 17 to 20 wt% aq. NaOH, see Figure 1. The R-value decreased to 0.8–1.0% units when increasing temperature from 40 to 50 °C. This result was contradictory to general understanding, but in accordance with the results presented in Fechter et al. [12]. There, it was shown for three basically different dissolving pulps that increasing temperature led to lower R-value at alkali concentrations ≥14 wt% aq. NaOH. This trend was even more pronounced for the pulp with the highest xylan content. The xylan rich hardwood kraft dissolving pulp was comparable to the pulp used in this study. 

### 2.2. The Change in Purity of the Pulp Caused by Alkalization

**Figure 2:**
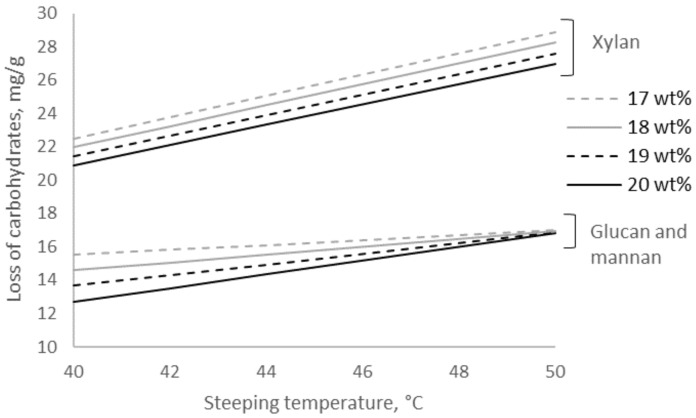
Modeled loss of carbohydrates in the residue after extraction of pulp with aqueous NaOH at different concentrations and temperatures.

Fechter et al., who have studied a wide span and a low resolution of extraction settings (10, 14, and 18 wt% aq. NaOH at 20, 35, and 50 °C), have shown the highest purity of xylan at relatively less loss of glucan, i.e., the highest selectivity, to be reached after extraction with high alkali concentration at high temperature (18 wt% aq. NaOH at 50 °C) [12]. The present study investigated these extraction settings in more detail and confirmed the finding that purification from xylan was most intense at 50 °C, which again was the highest temperature of the investigated span (Figure 2). The modeled data revealed a tendency for the highest selectivity for extraction of xylan to be at the lowest investigated alkali concentration (17 wt% aq. NaOH) at 50 °C. At 50 °C, the same amounts of glucan and mannan were modeled to be extracted independent of the alkali concentration applied. 

### 2.3. Molecular Weight

**Figure 3:**
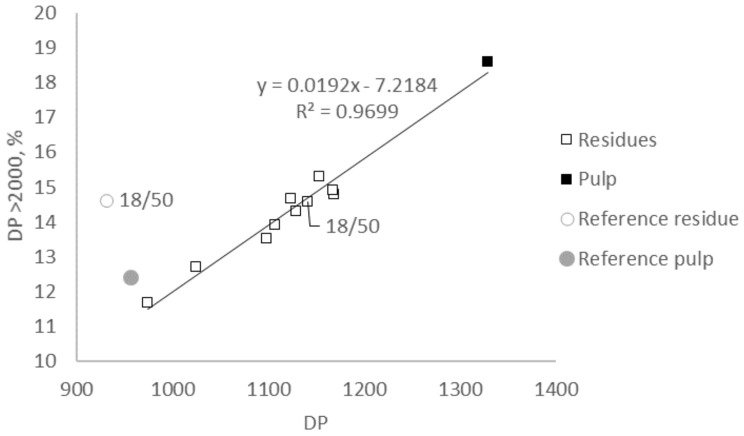
Correlation of degree of polymerization (DP) and fraction of material with DP > 2000 as determined with GPC-MALS after alkaline extraction of hardwood kraft dissolving pulp with 17–20 wt% aq. NaOH at 40–50 °C. The reference pulp (a hardwood kraft dissolving pulp with lower viscosity but identical chemical composition) and its residue after extraction with 18 wt% aq. NaOH at 50 °C is according to Fechter et al. [12].

The results obtained from GPC-MALS (gel permeation chromatography detected with multi-angle light scattering) allowed no statistically firm modeling of the influence of the factors of alkali concentration and temperature for the extractions done with 17–20 wt% aq. NaOH at 40–50 °C (Table 1). Interestingly, both the fraction of DP > 2000 and the DP decreased for all alkaline extraction settings applied. The decreases were found to be linearly positive correlated with R^2^ = 0.97 (Figure 3). These results were in contrast with the findings of Fechter et al. [12]. In that study, the investigated pulp, which differed from the investigated pulp in the present study by a lower DP, increased the fraction of high-molecular-weight material, while DP decreased slightly for the whole span of extraction settings applied (10–18 wt% aq. NaOH at 10–50 °C). The low-molecular-weight fraction decreased at the same time. The resulting polydispersity index (PDI) was almost stable with a tendency to decrease. In the present study, no general trend for these expected changes upon alkaline extraction, i.e., purification from low-molecular-weight material and decrease of the PDI, could be observed. This can be explained by the higher molecular weight of the sample in the present study. For a pulp with a low initial intrinsic viscosity, the purification from low-molecular-weight material will dominate the molecular structure after alkaline extraction (less low-molecular-weight material results in a larger fraction of high molecular weight and smaller PDI). For the actual pulp with a ca. 80 mL/g higher intrinsic viscosity, the deterioration of the high molecular weight dominated the molecular structure after alkaline extraction, with a smaller fraction of high-molecular-weight material in the residues. For low molecular weight material and PDI no clear trend was found. Most extraction settings led to a reduction of low-molecular weight fractions and the PDI. However, extraction with 17 wt% aq. NaOH at 44 °C and 20 wt% aq. NaOH at 50 °C led to an increase in both low-molecular-weight fraction and PDI compared to the properties in the initial pulp sample. No explanation was found for these results, but it can be concluded that the higher alkali concentration (≥18 wt% aq. NaOH) in combination with the lower temperature span (≤44 °C) resulted in the highest decrease of PDI.

### 2.4. Transformation of Cellulose I to Cellulose II

The degree of transformation to alkali cellulose (Na-Cell) can be determined by analysis of the content of cellulose II (Cell II) before and after alkalization and regeneration. This is because native cellulose exists as the specific lattice cellulose I (Cell I). This lattice is still the main fraction of the crystalline fraction in the pulp investigated (Table 1). When washing and neutralizing Na-Cell, and thus removing NaOH, regeneration into the thermodynamically more stable crystal lattice of Cell II takes place [20,21]. The degree of transformation gives information on the resistance of a specific pulp to alkalization. The accuracy of the determination of the degree of transformation with Raman spectroscopy as introduced by Fechter et al. was assumed to be 5% [12], and no differences between the extractions done with 17–20 wt% aq. NaOH at 40–50 °C could be stated in the present study. The alkaline treatments led to a degree of transformation of ca. 86% as the 95% confidence interval for the mean conversion of all samples was 86.3 ± 2.5%. This degree of transformation corresponded to the transformation for that kind of pulp under comparable conditions reported by Fechter et al. [12].

## 3. Discussion

**Figure 4:**
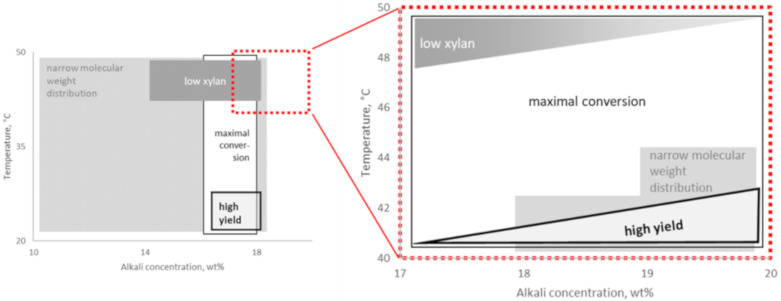
Trends for the key parameters of yield, xylan content, conversion to alkali cellulose and molecular weight distribution for different alkaline extraction settings of dissolving pulp. The illustration of the wide span of settings to the left is according to Fechter et al. [12].

This study gave both reliable and logical results when investigating the narrow range of alkalization settings used in industrial processes (17–20 wt% aq. NaOH at 40–50 °C). The trends shown for the settings when optimizing toward low xylan, high yield, and maximal conversion to alkali cellulose (Na-Cell) both complement and develop the findings in Fechter et al. [12] (Figure 4). Low xylan content and high yield could be shown to be contradicting within the applied narrow range of extraction settings with primarily low temperature and secondly high alkali concentration favoring yield. They varied to an extent that makes case-adapted process optimization meaningful. 

**Figure 5:**
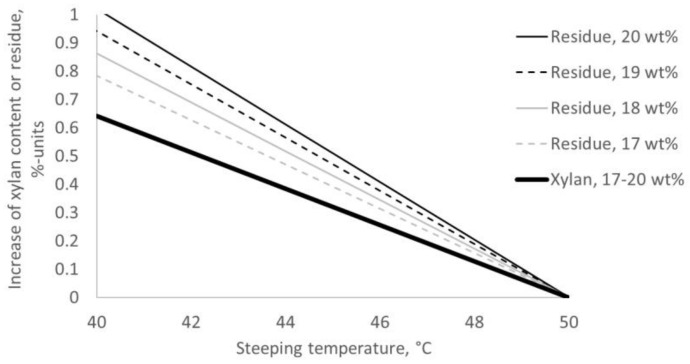
Change in xylan content and residue after extraction of hardwood kraft dissolving pulp with 17–20 wt% aq. NaOH when decreasing the steeping temperature from 50 to 40 °C.

**Figure 6:**
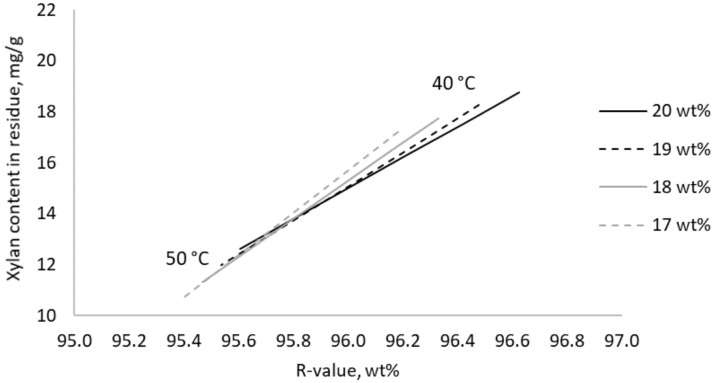
Modeled correlation between residue after extraction of hardwood kraft dissolving pulp with aqueous NaOH at different concentrations and temperatures, expressed as R-value, and the xylan content left in its residue.

Best selectivity was found after extraction with low alkali concentration at the high temperature of 50 °C. At the same time, yield was at a minimum within the investigated range of extraction settings. A decrease of the steeping temperature by 2 °C when extracting with 17 wt% aq. NaOH increased yield with 0.15% units, whereas extracting with 20 wt% led to an increase of 0.20% units (Figure 5). When optimizing for higher yield by extracting at lower temperature, higher alkali concentration resulted in a lower xylan content in the residue at a given yield as residue (Figure 6). 

The finding that the yield-favoring conditions, higher alkali concentration and lower extraction temperature, resulted in a narrower molecular weight distribution (lower PDI) for the specific pulp investigated was unexpected. In any case, the hypothesis that alkaline extraction parameters matter for the resulting distribution of different molecular weight fractions could be neither refuted nor confirmed. For further research work, it is assumed that the initial molecular weight and its distribution in the pulp might play an important role for the resulting distribution of molecular weight fractions in the residue. Consequently, it would be of interest to further investigate how the initial molecular weight in a pulp influences the distribution of different fractions after alkaline extraction.

## 4. Materials and Methods 

### 4.1. Material

Industrial dried hardwood kraft dissolving pulp was provided by a market pulp producer. Acetic acid 95–97%, aq. NaOH p.a., water-free sodium acetate (CH_3_COONa) p.a., L(+)-arabinose 99%, D-xylose 99%, D-(+)-mannose 99%, D-(+)-galactose 99%, D-(+)-glucose 99.5%, HCl p.a., copper ethylene-diamine solution (1.00 mol/L), LiCl, ethanol p.a., and *N*,*N*-dimethylacetamide, 99.9+ % were purchased from Sigma Aldrich (Stockholm, Sweden).

### 4.2. Methods

#### 4.2.1. Preparation of Residues (R) and Determination of the R-Value

Regenerated alkali celluloses as residue (R) after alkaline extraction of the pulp, replacement washing and subsequent oven drying were produced according to the procedure for the determination of alkali resistance of pulp expressed as R-value (ISO 699:2015) using 17–20 wt% aq. NaOH at 40 to 50 °C. As the standard procedure uses aqueous alkali at 20 °C, the procedure had to be modified to compensate for the lower viscosity of the warmer alkali solution. When applying temperatures > 40 °C, a finer glass filter (40–100 µm) had to be used.

Extraction experiments according to the scheme given in Table 2 were conducted. All settings, despite the extraction with 18 wt% aq. NaOH at 42 °C, were repeatedly determined. Modde12.1 (Umetrics/Sartorius, Göttingen, Germany), and JMP^®^14.0 (SAS Institute, Cary, NC, USA). were used as modeling tools.

#### 4.2.2. Analysis of Pulp and Its Residues

The carbohydrate composition was analyzed via acid hydrolysis (TAPPI—T 249 cm-85) using 72 wt% aq. H_2_SO_4_. The liberated neutral monosaccharides in the hydrolysis filtrate were quantified using High-Performance Anion-Exchange Chromatography (HPAE ICS-3000 from Dionex (Sunnyvale, CA, USA) with Pulsed Amperometric Detection (PAD)) and reported as anhydrous carbohydrate according to SCAN-CM 71:09.

Intrinsic viscosity (limiting viscosity number) was analyzed according to ISO 5351:2010 based on the dissolution of the sample in copper ethylene-diamine solution (CED).

Molecular weight distribution was determined using GPC-MALS (solvent DMAc/LiCl) according to Henniges et al. on the following equipment [22]: an RI detector Optilab T-rEX (Waytt, Santa Barbara, CA, USA), a Multi Angle Light Scattering Detector Dawn Heleos II (658 nm) (Waytt, Santa Barbara, CA, USA) and four columns (photoluminescence gel mixed A LS, 0.20 lm, 7.5 9 300 mm) from Agilent Technologies (Santa Clara, CA, USA). Data processing was done in Wyatt’s Astra6 to calculate key values such as the weight-average degree of polymerization (DPw, DP), the polydispersity index (PDI), and the weight fraction of molecules with DP < 100.

NIR FT Raman spectroscopy was performed using a MultiRam III (Bruker, Billerica, MA, USA) with a liquid-nitrogen-cooled Ge diode as the detector. A Nd:YAG laser (1064 nm) with a maximum power of 500 mW was the light source for the excitation of the Raman scattering. Because of the solid and compact structure of the residues, no further sample preparation was necessary. A total of eight points per sample was measured. Each point was measured 100 times. The data were collected and analyzed using OPUS software (Cooperative Library Network Berlin-Brandenburg, Germany). All spectra of one sample were combined into one, and this averaged spectrum was then handled with OriginPro (OriginLab, Northampton, MA, USA). Cellulose II was calculated according to Agarwal and the degree of transformation according to Fechter et al. [12,23].

## Figures and Tables

**Table 1:** Properties of the hardwood kraft dissolving pulp (-) investigated and experimental data for the residues after alkaline extraction at different alkali concentrations of aqueous NaOH and temperatures.

NaOH- Conc.	wt%	-	17	17	18	18	18	18	19	19	20	20
Extraction Temp.	°C	-	40	44	40	42	44	50	42	44	40	50
Residue ^1^	wt%	-	96.1	95.8	96.5	96.2	96.0	95.5	96.2	96.1	96.6	95.6
Glucan ^2^	mg g^−1^	953	978	980	976	977	979	983	978	978	976	982
Xylan ^2^	mg g^−1^	39	17	14	18	17	15	11	17	16	18	13
Mannan ^2^	mg g^−1^	4	2	2	2	2	2	2	2	2	2	2
Mn	kg mol^−1^	65	57	49	72	71	61	65	68	71	66	46
Mw	kg mol^−1^	215	182	166	178	189	187	185	183	189	179	158
Mz	kg mol^−1^	466	371	355	332	374	367	355	353	369	334	322
PDI ^3^		3.3	3.2	3.4	2.5	2.7	3.1	2.8	2.7	2.7	2.7	3.4
DP ^4^		1327	1123	1025	1099	1169	1153	1141	1130	1167	1107	975
DP < 50	%	0.9	1.0	1.6	0.2	0.4	1.0	0.7	0.4	0.4	0.5	1.4
DP < 100	%	2.8	2.7	3.7	1.2	1.6	2.5	2.0	1.6	1.6	1.8	3.6
DP > 2000	%	18.6	14.7	12.7	13.5	14.8	15.3	14.6	14.3	14.9	13.9	11.7
Degree of Transformation ^5^	%	-	86	88	88	85	87	86	85	86	88	85

^1^ The real pooled difference for the residues after alkaline extraction in this study is 0.08%-units, to compare with 0.3%-units given in the standard procedure; ^2^ the saccharides are reported as pure anhydrous polysaccharides; ^3^ polydispersity index, determined with GPC-MALS (gel permeation chromatography detected with multi-angle light scattering); ^4^ weight average degree of polymerization, determined with GPC-MALS; ^5^ parameter of cellulose structure measured with Raman spectroscopy and evaluated according to Fechter et al. as a fraction of crystalline cellulose [12].

**Table 2:** Trial scheme for preparation of residues for further investigations.

	17 wt% aq. NaOH	18 wt% aq. NaOH	19 wt% aq. NaOH	20 wt% aq. NaOH
40 °C	X	X		X
42 °C		X	X	
44 °C	X	X	X	
50 °C		X		X
